# The Relations of Hospitals to Public Health

**Published:** 1893-07-15

**Authors:** John S. Billings

**Affiliations:** being his Address as Chairman of the Section on Hospital Care of the Sick, &c, International Congress of Charities, Chicago


### The Relations of Hospitals to Public Health.

By John S. Billings, M.D., being his Address as Chairman of the Section on Hospital Care of the Sick, &c., International Congress of Charities, Chicago.

The business of this section relates to co-operative means for the care of those suffering from disease or injury, more especially of recent origin, excluding for the most part those forms of brain abnormity or disease connected with what are known as insanity and idiocy, and also those forms of chronic and incurable disability which are not amenable to medical and surgical treatment.

Primarily, hospital aid was intended, and provided solely for the benefit of the poor; of those who were unable to obtain at their own expense, or by their own efforts, proper care in case of sickness; but its field of work has been steadily extending, and it now has relations with the interests of almost every class of the community, and its results have greatly modified the methods of treatment of many forms of disease among the well-to-do classes, as well as among the poor.

It is largely by hospital organisation and work that skilled physicians, surgeons, and nurses are provided for the public, and in the absence of hospitals their proper and complete training is practically impossible.

Each succeeding year more people resort to hospitals and dispensaries for treatment, and this is especially the case in the United States. Forty years ago the number of hospital beds in our cities was very small in proportion to the population, when compared with the amount of such accommodation in the countries of Eastern Europe, and the demand for such accommodation was also small. People did not go to hospitals if they could help it; it was believed that surgical operations and labour cases did not result so well in hospitals as they did in the homes of the people, even when these homes were very small and not specially well ordered. Hospitals were for sick paupers, and we did not have many paupers in comparison with European countries. The war of 1861-65, and the great influx of immigrants have produced many changes in public opinion upon these points.

The war taught us how to build and manage hospitals so as to greatly lessen the evils which had previously been connected with them, and it also made the great mass of the people familiar with the appearance of and the work in hospitals as they had never been before. Not only the tens of thousands of men who were treated in the great war hospitals in those days, but the hundreds of thousands of visitors, the parents, children, sisters, and friends of these men were thus educated, and they were educated not merely by what they saw and heard, but by what they did or tried to do to help make the patients more comfortable, by the work of the sanitary and Christian commissions, by the formation of local associations for giving aid and relief, and by becoming accustomed to the methods and results of sanitary co-operation in matters of this kind.

Since the close of the war the formation of training schools for nurses in many of the large hospitals has been an additional means of interesting the public in the work, and of keeping it informed as to the progress made in securing the safety and comfort of the inmates. With the increase of knowledge about hospitals and their capabilities has come an increased demand upon them for accommodation for persons in comfortable circumstances who are affected with diseases which can be better treated in them than in private houses; in other words, for private rooms for pay patients, especially those requiring surgical operations, or suffering from certain forms of nervous diseases; and from this class of persons and their friends the demand is now relatively greater in the United States than it is in Europe.

During the last thirty years the demand for free beds in the public wards has also greatly increased, owing in part to a relative increase in the number of the very poor in our large cities, and in part to the immigration of large numbers of people, accustomed in their former homes to seek public aid and hospital relief in case of sickness, and bringing with them this habit, which extends to others by force of example.

The increase in free dispensary work, or out-patient relief, as it is sometimes called, has been even greater than that in free hospital beds, or in-patient relief in our large cities, and the number of people who are not paupers who apply to these dispensaries for free treatment, although they are able to pay reasonable fee if required to do so, is becoming so large as to constitute a serious problem in hospital and dispensary management with us, as it does in London and other large cities.

As the health of a community depends on the health of the individuals who compose it, it is evident that there may be important relations between hospital aid in all its aspects and the public health, and it is to some of these relations that I desire briefly to call your attention.

The importance of hospitals for certain forms of contagious and infectious disease, as a means of preventing the spread of such diseases, would appear to be almost self-evident, yet very few cities in this country are provided with them. If there is a city "pest house," as it is commonly called, it is the relic of a small-pox outbreak, and is usually empty and uncared for, located in some desolate suburb, the grounds overgrown with weeds, and the building itself corresponding in appearance to the ideas to which its name naturally gives rise.

We have several papers before the Section on the subject of hospitals for infectious disease, and it is therefore unnecessary for me to say anything about the plans and arrangements for such institutions, which will, no doubt, be fully discussed in the Section meetings; but there is one point with regard to them to which I will briefly refer, namely, the question as to whether they should, or should not, be entirely free to all persons, no matter whether they are able to pay for the accommodation provided or not. It is urged by the majority of English health officers that the isolation of a case of infectious disease in a special hospital provided by the sanitary authorities for that purpose, is not, in most cases, any special benefit to, or favour conferred upon, the person so treated; that it is done, and made compulsory, for the benefit of the community and not of the individual, and that the community should, therefore, bear the cost. It appears to me that this is true with regard to necessary cost, and to that only. It is not only permissible, but desirable, that such a hospital should be able to furnish a private room, a special nurse, and other extra accommodations, when demanded, as they would be if such hospitals were made use of by well-to-do people, and the party receiving such extra service and accommodation should pay for it.

To make such hospitals really useful in preventing the spread of disease, there should be the least possible delay and formality in admitting cases. If a child affected with scarlet fever or diphtheria is brought to the door, and the medical officer recognizes it to be such, it should be admitted at once, without waiting to send for a permit from some official; and the general rule should be that a certificate from any competent physician that the person is suffering from such a disease as the hospital is intended for, should be a sufficient warrant for his admission.

The increasing use of hospitals and free dispensaries to which I have referred, is one of the signs of the socialistic tendency of the age, of the increasing tendency to subordinate the individual to the community in attempting to equalise the burdens and pleasures of mankind. If the process be carried a little further, we might come to something like the scheme suggested by Mr. Havelock Ellis in his recent book, entitled "The Nationalisation of Health." This is to the effect that the hospitals of the future are not to be charitable or voluntary institutions, but are all to be under national control, to be supplied from national funds, and to be free to everyone. The country is to be covered with a network of such hospitals, each having a large medical staff, including all sorts of specialists paid by the State. Private practitioners are no longer to be relied upon for medical attendance to the public; it is supposed that they would only be consulted for minor and comparatively trivial ailments; the greater part of the work is to be done by medical officials. Private charity and individual philanthrophy are no longer to be relied upon or encouraged; the whole business is to be done by machinery; health is to be equalised among the people. All hospitals are to be placed on the footing of hospitals for contagious diseases, and, with their medical staffs, are to become a part of a greater national bureau for the prevention of disease. As it is to the interest of a medical officer of the army or navy to prevent, as far as possible, the occurrence of disease among the command to which he is assigned, in order that he may have as little as possible to do in the way of treatment, so it is supposed that these other medical officials will be active, zealous, and efficient agents in prescribing and enforcing State and municipal sanitation.

Two hundred and fifty years ago Sir Thomas Browne said that he counted this world not an inn but an hospital, a place, not to live, but to die in, and perhaps the plan I have outlined, when fully carried out, will make many men of his way of thinking. I do not myself think that this scheme will be carried out, but I do think that the present tendency is in that direction, that hospital aid will be more and more resorted to in coming years, that there will be an increasing demand on the part of the constituted authorities representing the majority of the people for State or municipal supervision of what are at present private charities, upon grounds similar to those stated by Mr. Ellis, and that it is important for us to recognise these facts and tendencies, whether we approve of them or not. I myself think that hospitals supported by voluntary contributions confer quite as much benefit upon those who contribute the funds as upon those who are treated in them. If, however, there is to be public and official supervision of all free hospitals and dispensaries, should not these be in closer relation with, and contribute more to the public health service of the cities in which they are placed than is the case at present? Even the city hospitals, those that are supported entirely by municipal funds, do not, as a rule, have any special connection with the city health department, but are under entirely different management. They report the deaths which occur in them, and sometimes the cases of certain forms of contagious disease which are treated in them, but little more. As to the voluntary hospitals and dispensaries that are supported from private funds, they make the same sort of reports and nothing more. But if my view of the tendencies of the age is correct, the time is not far distant when the health office of a city will have a daily record, not only of all deaths, but of all cases of disease treated free in any hospital or dispensary in the city, with specifications of name, age, sex, colour, place of residence, nature of disease, and mode of final disposal. I need hardly comment on the value of such a record, both as an immediate emergency guide for the health officer, and as a basis for statistical investigation of the healthfulness of different parts of the city.

I have already indicated the important relations to public health held by hospitals in their function of aiding in the training of physicians and nurses, and this a point which should be constantly borne in mind in attempts to compare the efficiency and economy of different hospitals, or of the same hospital at different times. The teaching hospitals not only do the best work in the treatment of patients who are more carefully examined and more scrupulously cared for in them than they are in non-teaching institutions, but they furnish the doctors and nurses required by the people in their own homes, and the quality of their work in this respect merits more scrutiny on the part of the public than it has heretofore received.

No doubt it would be a new idea to our mayors and municipal authorities if they were told that they are, to a considerable extent, responsible for the quality of teaching and the standards for graduation in those medical schools which obtain their facilities for clinical instruction in the hospitals supported by the city; yet it is the truth, since they have it in their power to enforce almost any standard of medical education which they choose to favour.

The tendency in this country is, however, towards the regulation of standards of medical education by the State, and some curious questions of jurisdiction will arise if a State should undertake to prescribe the conditions under which instruction in practical medicine and nursing shall be given in the hospitals of its several municipalities.

At present our best medical schools are desirous of having hospitals of their own, or, at all events, in which they can control the appointments of the attending and resident medical staff, since, otherwise, the selection of some of their clinical teachers may be made by those who have no interest in or responsibility for the work of the school. For the same reason the establishment of a large general hospital by private endowment presents a strong inducement to the establishment of a medical school closely connected with it and under the same control—in fact, such a hospital without a medical school, and nurses' training school, is only doing a part of the work which is rightfully expected and demanded of such an institution.

To a limited extent this obligation to promote the public health by increasing and diffusing knowledge as to the causes, nature, and best methods of prevention or treatment of disease, rests also upon special hospitals, including those for the insane, and no such hospital can be considered as doing its complete and best work if it is not contributing to training of physicians and nurses.

With the present rapid concentration of population in cities, the demand for hospital accommodation will steadily increase, and so also will the demand for municipal regulation of dwellings in their sanitary aspects, for increase of facilities for limiting the spread of contagious and infectious disease, and for skilled supervision of food supplies. All these things are more or less correlated, and they should be studied together.

I do not think that any millennium will thus be produced, or that Nature's methods of eliminating the idle, the vicious, and the unfit by diminishing birth rates and increased death rates will be either rendered unnecessary, or done away with, by advances in medicine or in sanitation; but whether this be true or not, it is clearly the duty of those who have knowledge, means, and opportunities, to investigate these matters carefully, and to do all that they can to lessen the sufferings and sorrows of those who are unable to help themselves.

